# Neural circuit mechanisms of acupuncture effect: where are we now?

**DOI:** 10.3389/fneur.2024.1399925

**Published:** 2024-06-13

**Authors:** Xuesong Wang, Jia Wang, Rui Han, Chaochao Yu, Feng Shen

**Affiliations:** ^1^College of Acupuncture-Moxibustion and Tuina, Hebei University of Chinese Medicine, Shijiazhuang, China; ^2^Department of Acupuncture and Moxibustion, Wuhan Hospital of Integrated Traditional Chinese and Western Medicine, Wuhan, China; ^3^Department of Child Rehabilitation Medicine, Qujing Hospital of Maternity and Childcare, Qujing, China; ^4^Department of Tuina, Shenzhen Traditional Chinese Medicine Hospital, Shenzhen, China; ^5^The Fourth Clinical College of Guangzhou University of Chinese Medicine, Shenzhen, China; ^6^Department of Rehabilitation, Union Hospital, Tongji Medical College, Huazhong University of Science and Technology, Wuhan, China; ^7^College of Acupuncture and Orthopedics, Hubei University of Chinese Medicine, Wuhan, Hubei, China

**Keywords:** acupuncture, neural circuit mechanisms, pain, Parkinson’s disease, addictive disorders, cognitive disorders, gastrointestinal disorders, review

## Abstract

Recently, there has been increasing attention on the impact of acupuncture on the dysregulated neural circuits in different disease. This has led to new understandings of how acupuncture works. This review presents a comprehensive analysis of research that have examined the impact of acupuncture on abnormal neural circuits associated with pain, anxiety, Parkinson’s disease, addiction disorders, cognitive problems, and gastrointestinal disorders. These studies have shown that acupuncture’s therapeutic effects are mediated by specific brain areas and neurons involved in neural circuit mechanisms, emphasising its wide-ranging influence. The positive impacts of acupuncture can be ascribed to its ability to modify the functioning of neurocircuits in various physiological conditions. Nevertheless, contemporary studies on acupuncture neural circuits frequently overlook the comprehensive circuit mechanism including the periphery, central nervous system, and target organ. Additionally, the scope of diseases studied is restricted. Future study should focus on broadening the range of diseases studied and exploring the neural circuit mechanisms of these diseases in depth in order to enhance our understanding of acupuncture’s neurobiological impacts.

## 1 Introduction

Acupuncture has a rich historical background and holds significant cultural importance in China, it is an important part of traditional Chinese medicine (TCM). Acupuncture originated in ancient China and has been developed and inherited over thousands of years of history. In the ancient medical literature of China, such as the Miraculous Pivot (《灵枢》), there are numerous documented instances of acupuncture.

Currently, acupuncture remains extensively utilized in China and is progressively garnering global interest and acknowledgment. Acupoints, according to traditional meridian theory, are the convergence points of *Qi* (气, vital energy), blood, and meridians within the internal *Zang-Fu* (脏腑). They are also reflective areas on the body surface corresponding to the internal *Zang-Fu* (脏腑). Acupoints play a crucial role in the therapeutic effectiveness of acupuncture. Meridians have many functions, including connecting internal *Zang-Fu* (脏腑), communication between internal and external, responsible for the operation of *Qi* (气, vital energy) and blood, nutritional *Zang-Fu* (脏腑), defense against disease, balance of *Yin* (阴) and *Yang* (阳). The functioning of life activities in the human body is governed by *Qi* (气, vital energy), and acupuncture can be employed to regulate the functioning of *Qi* (气, vital energy) and blood by stimulating certain acupoints, so achieving the objective of healing diseases and maintaining health.

In recent years, the field of neuroscience has made significant progress in enhancing our comprehension of the neural circuits in organisation and operation of brain networks. “Neural Circuits” consist of neurons and glial cells (especially astrocytes and microglia), and are the basic units in the nervous system for transmitting and processing neural signals. Neural circuits play a crucial role in overseeing and managing a wide range of physiological functions in the body, including perception, movement, cognition, and emotion. Acupuncture has been discovered to have the ability to regulate the physiological activities of the organism and generate therapeutic effects by influencing various neural circuits and neurotransmitter systems. Hence, the study of neural circuits is essential for comprehending the mechanisms and impacts of acupuncture, while also offering novel concepts and directions for advancing the field of acupuncture. With the ongoing advancement of acupuncture standardization, more and more high-quality clinical and basic evidence of acupuncture is being reported, and the recognition of acupuncture is increasing internationally (1). This has led to a rising international acknowledgment of acupuncture. Modern medical research has shown that acupuncture exerts therapeutic effects through mechanisms such as anti-inflammatory effects (2), immunomodulation (3), regulation of synaptic plasticity (4), and anti-apoptotic processes (5). In recent years, acupuncture related neural circuit studies have become the focus of growing research interest, mainly in the areas of pain (6), pain-related anxiety (7), Parkinson’s disease (8), addiction (9), cognitive disorders (10), and gastrointestinal disorders (3). Here, we provide a review of current research with acupuncture-related neural circuit studies according to different diseases, and our objective is to provide new perspectives and a deeper understanding of how acupuncture works.

## 2 Pain and pain-related anxiety

Pain is a physiological response of the body to potential injury or damage to tissues, manifested by way of nerve signaling. The condition can manifest as either acute, meaning it develops rapidly and subsides fast, or chronic, lasting for more than three months. Pain can arise from a range of factors, such as tissue damage, chronic diseases, and neurological abnormalities (11). Furthermore, pain is not solely a physiological occurrence, but is intricately connected to psychological and emotional reactions. Chronic pain is frequently accompanied by emotional disorders like anxiety, which can worsen the experience of pain and possibly impacts the effectiveness of treatment (12, 13). There have been numerous research conducted on the analgesic effects of acupuncture (14–17). Research in classical studies has demonstrated that acupuncture has the ability to stimulate the release of occurring opioid peptides, such as enkephalin and dynorphin. This stimulation also activates neural activity in the dorsal region of the grey matter surrounding the aqueduct, resulting in analgesic effects within the central nervous system (18, 19). Furthermore, acupuncture has the ability to regulate the secretion of pain-related molecules, including dopaminergic and serotonergic neurotransmitters (20), acetylcholine (21), ATP/adenosine (22), purinergic signaling (23). It also has the capacity to suppress the release of pain-inducing substances, hence alleviating pain.

Acupuncture is essential in regulating pain at the spinal level. During pathological conditions, acupuncture sites can have an impact on the circuits that transmit pain. Pain caused by harmful stimuli usually includes receptors located in the peripheral nerves. These receptors convey signals through the spinal nerve roots to the dorsal root ganglia (DRG), and eventually reach the thalamus and other brain structures (24). As primary afferent neurons, pain signals travel through C fibers or Aδ fibers in the DRG to the spinal cord. Studies have investigated the neurobiological processes of acupoints sensitization that are associated with the activation of primary afferent nerves (25). Studies indicate that C fibers in the DRG are implicated in acupoints sensitization (26). Further research, retrograde tracing and chemogenetics, revealed that the Zusanli (ST36) acupuncture point and the corresponding plantar area on the same side of the body had shared nociceptive dorsal root ganglia (DRGs). Alterations in the sensitivity of these commonly shared nociceptive dorsal root ganglia (DRGs) are associated with changes in the thresholds for mechanical and thermal pain at the Zusanli (ST36) acupoint in a mouse model treated with complete Freund’s adjuvant (CFA). This indicates that the overstimulation of the shared DRGs at the site of injury and the corresponding acupoint plays a role in the development of increased sensitivity to acupuncture. Furthermore, Zhu (27) found that acupoints not only generate sensitization signals but also communicate information to the central nervous system. This process activates the endogenous pain modulation system through descending inhibitory pathways, resulting in systemic analgesic effects. The endogenous pain modulation system encompasses modulation at multiple levels, including the spinal level, central nervous system, brainstem, and forebrain. The descending inhibitory system is essential for regulating pain and involves structures such as the periaqueductal grey (PAG), rostral ventromedial medulla (RVM), locus coeruleus (LC), and lateral reticular nucleus (LRN) (28). The interconnectedness of these tissues is facilitated by descending pathways that control the activation of primary afferents in the spinal dorsal horn. One of these components, the periaqueductal grey (PAG), serves as a central hub by engaging in the majority of higher-level central pain inhibitory pathways and triggering the descending inhibitory system. Acupuncture can alleviate pain by activating certain internal pain control structures or activating neuronal pathways in the inhibitory system that reduces pain sensation (29).

The thalamus functions as an intermediary in the transmission of pain signals, playing a crucial role in facilitating the passage of nociceptive information to the cortex. The thalamus is divided into three parts: the posterior, medial, and lateral thalamus. Each of these divisions receives signals related to pain from the spinal cord (30). The posterior thalamus primarily projects to the somatosensory cortex (SSC), contributing to the discrimination of pain sensation. Neurons in the medial thalamus not only project to the motor areas of the cingulate gyrus but also regulate the anterior cingulate cortex (ACC) and prefrontal cortex, thereby participating in the emotional and attentional modulation of pain perception. The lateral thalamus predominantly projects to the dorsolateral prefrontal cortex (DLPFC), engaging in the cognitive evaluation of pain (31). Acupuncture exerts regulatory effects on pain sensation, emotion, and cognition by modulating neural circuits within the thalamus and cortex (32). Acupuncture therapy for chronic pain may involve the regulation of GABAergic neurons surrounding the ventrolateral periaqueductal gray (vlPAG) via various pathways, thereby attenuating or relieving 5-HT-related descending inhibitory control and augmenting the pain-modulating function of the descending inhibitory system (33). Researchers have observed that enhanced expression of cannabinoid cannabinoid receptor 1 (CB1) on midbrain GABAergic neurons can alleviate 5-HT-related descending inhibitory control and diffuse noxious inhibitory control (DNIC) function, consequently alleviating chronic pain (34). In a mouse model of knee osteoarthritis (KOA), low-frequency, high-intensity electroacupuncture at the Zusanli and Sanyinjiao points enhances the expression of CB1 receptors on midbrain GABAergic neurons by activating CB1 receptors in the ventrolateral periaqueductal gray (vlPAG), thereby eliciting the aforementioned effects, indicating that acupuncture may alleviate chronic pain by regulating the expression of ventrolateral periaqueductal gray (vlPAG) CB1 receptors to enhance the descending inhibitory system (35). Similarly, researchers have noted the potential influence of the vlPAG’s activating effect on the descending inhibitory system on pain-related negative emotions in the rostral anterior cingulate cortex (rACC) (36). Researchers found that GABAergic neurons in the vlPAG receive glutamatergic projections from the rostral anterior cingulate cortex (rACC), and inhibiting the Glu-vlPAG circuit in the rostral anterior cingulate cortex (rACC) leads to feedforward inhibition of 5-HT neurons by GABAergic neurons in the vlPAG (37). Activating this circuit in spared nerve injury (SNI) model mice can counteract the analgesic effect of electroacupuncture but cannot reverse its anxiolytic effect, suggesting that electroacupuncture may alleviate neuropathic pain hypersensitivity through this neural circuit rather than its anxiolytic effect (38). Furthermore, Wu et al. (39) demonstrated that electroacupuncture alleviated chronic pain-induced anxiety-like behaviors via activation rACC^CaMKII^-DRN^5-HT^ circuit. Shen et al. (38) found that electroacupuncture can treat anxiety-like behavior in Complete Freund’s Adjuvant (CFA) model rats by activating the rACC Glu-thalamus circuit without affecting pain. Xu et al. (40) found that electroacupuncture can exert analgesic and anxiolytic effects on spared nerve injury (SNI) model mice by activating the rACC Glu-dorsal raphe nucleus (DRN) circuit. Hsiao et al. (41) found that electroacupuncture to the Zusanli (ST36) could treat chronic inflammatory pain by inhibiting CaMKIIα signaling in the somatosensory cortex (SSC)-anterior cingulate cortex (ACC) circuit.

The limbic and reward systems are crucial brain regions linked to the control of emotions, motivation, and pain. The limbic system comprises the hypothalamus, amygdala, and hippocampus, primarily responsible for emotional regulation, memory formation, and stress reactions in response to pain. Concurrently, the reward system, comprising the nucleus accumbens (NAcc), ventral tegmental area (VTA), and substantia nigra, is believed to have a strong connection to several elements of pain perception, such as pleasure and addiction. Additionally, it plays a role in broader reward processing. The interplay between these systems impacts an individual’s perception and emotional reaction to pain, therefore influencing their capacity to manage and adjust to pain (42). Acupuncture can modulate the emotional and motivational aspects of pain by influencing neural circuits within the limbic and reward systems, while also impacting the rewarding effects of pain. Recent studies have shed light on the role of the lateral hypothalamus (LH) in regulating neuropathic pain (43). The study found that LH neurons projecting from the lateral habenula (LHb) are involved in modulating neuropathic pain (44). These discovered that GABAergic neurons in the lateral septum (LS) projecting to the lateral hypothalamus (LH) are implicated in the comorbidity of pain and anxiety. These findings suggest that lateral hypothalamus (LH) may serve as a crucial center for pain modulation, and acupuncture may exert therapeutic effects on pain and anxiety by influencing lateral hypothalamus (LH) neural circuits (45). The nucleus accumbens (NAcc) is a central component of the brain’s reward system, primarily involved in dopamine-mediated reward and pleasure behaviors, which also has implications for the rewarding effects of pain (46). The rewarding effects of pain refer to the pleasurable or satisfying sensation experienced when pain is relieved or alleviated, thus increasing pain tolerance and acceptance. Acupuncture can influence the rewarding effects of pain by activating neural circuits within the nucleus accumbens (NAcc). Wang et al. (47) found that signals from acupuncture points travel from the spinal cord to the hypothalamus, and electroacupuncture at bilateral Zusanli (ST36) in spared nerve injury (SNI) model rats can induce conditioned place preference (CCP) during the early stages of chronic pain. Activation of the orexinergic neural circuit from the lateral hypothalamus (LH) to the nucleus accumbens (NAcc) shell induced by electroacupuncture-induced pain relief elucidates acupuncture’s potential to exert rewarding effects in alleviating chronic pain through this neural pathway (47).

In conclusion, acupuncture, as a pain management technique, engages numerous neural circuits and offers promising therapeutic benefits for a range of pain and pain-related mental disorders.

## 3 Parkinson’s disease

Parkinson’s disease (PD) is a common neurodegenerative disease of the central nervous system (48), which is characterized by significant degeneration and loss of dopamine (DA) neurons in the substantia nigra (49). The primary symptoms of PD include bradykinesia, tremor in the static state, emotional abnormality (mainly depression, anxiety and apathy), and postural instability (50). Acupuncture is a therapeutic method that can improve symptoms of Parkinson’s disease. When used alongside medication, it helps reduce side effects and increase effectiveness (51). The mechanism behind acupuncture’s treatment for PD may involve reducing oxidative stress, alleviate immune-inflammatory reactions, and regulate the gut-brain axis (52, 53).

Early research and anatomical studies have revealed that glutamatergic neurons in the cortex and thalamus, as well as dopaminergic neurons in the midbrain, project to the basal ganglia’s primary input nucleus—the striatum (54). Loss of dopaminergic neurons in the substantia nigra results in insufficient dopamine projection to the thalamus and cerebral cortex, leading to impaired motor control function in the basal ganglia circuitry (55). The inability of the substantia nigra to coordinate the movements of agonist and antagonist muscles gives rise to motor symptoms such as resting tremors in PD. Hence, the loss and degeneration of dopaminergic neurons play a crucial role in PD’s motor symptoms (56). Additionally, in animal models of PD, dopaminergic pathways in the basal ganglia, thalamus, and limbic system are also compromised (57). Functional magnetic resonance imaging (fMRI) can reveal changes in brain neural activity signals induced by acupuncture, aiding in the identification of brain regions involved in the neural pathways of acupuncture treatment for PD in clinical research (58). Chae et al. (59) treated 10 PD patients with acupuncture at the left Yanglingquan (SP9) acupoint, resulting in improvements in hand motor function, as indicated by fMRI activation of the thalamus and primary motor cortex post-treatment. Similarly, Yeo et al. (60, 61) demonstrated increased signals in PD-affected brain regions such as the substantia nigra, striatum, prefrontal cortex, and anterior cingulate cortex following acupuncture at the right Yanglingquan acupoint. These findings suggest that acupuncture may alleviate PD motor symptoms by activating PD-affected brain regions and modulating the basal ganglia-thalamocortical and cortical circuits. Li et al. (62) showed that acupuncture at Baihui, Fengchi, and the tremor area improved tremor symptoms in PD patients, possibly by affecting the cerebello-thalamo-cortical (CTC) circuit. fMRI observed specific activation of the cerebellum, along with various changes in the thalamus and motor cortex. It is noteworthy that the inconsistency in the regional homogeneity of the motor cortex fMRI between the left and right sides may be due to handedness. In animal experiments, Jia et al. (63) found that electroacupuncture reduced the loss of dopaminergic neurons in the substantia nigra of unilateral medial forebrain bundle (MFB)-lesioned PD model rats, reversed the decrease in midbrain substance P levels and the increase in glutamic acid decarboxylase-67 (GAD 67) mRNA levels induced by the lesion, and significantly reduced abnormal movements in PD model rats. They also found that high-frequency electroacupuncture reversed the increase in midbrain GABA content and promoted motor coordination in MFB-injured rats (64). These studies suggest that electroacupuncture therapy may improve PD motor dysfunction by restoring the homeostasis of basal ganglia circuits and inhibiting excessive GABA output.

In summary, recent studies indicate that acupuncture predominantly regulates the activity of GABAergic neurons in the basal ganglia and is involved in specific neural pathways connecting the basal ganglia, thalamus, and brain. This modulation decreases the degeneration of dopaminergic neurons in the basal ganglia, enhances the activity of dopamine receptors, therefore leading to an improvement in aberrant motor symptoms in PD. Moreover, there is a correlation between thalamic dysfunction and non-motor symptoms of PD, such as rigidity and tremor. Acupuncture may enhance non-motor symptoms of PD by activating alternative pathways, such as the cerebello-thalamo-cortical (CTC) circuit. Subsequent investigations should delve further into the mechanisms by which these neural circuits operate in order to gain a more comprehensive understanding of the impact of acupuncture therapy on the treatment of PD.

## 4 Addictive disorders

Addiction disorder refers to an intense reliance on a substance or behavior, leading to an uncontrollable compulsion to use or engage in it. This reliance is typically accompanied by strong psychological urges and physical symptoms of withdrawal. Addiction disorders can be addictions to substances or drugs (e.g., drugs, alcohol, nicotine, etc.) or addictions to certain behaviors or activities (e.g., gambling, shopping, food intake, etc.) (65). Addiction disorders are considered to be complex disorders that are influenced by genetic, environmental, and psychological factors (66, 67). The primary emphasis of current research on addiction disorders is substance addiction. Acupuncture is regarded as a supplementary therapy for addiction problems, aiding in the reduction of withdrawal symptoms, alleviation of worry and dread, and promotion of bodily and mental equilibrium throughout the withdrawal process. It has the potential to control levels of neurotransmitters, mitigate withdrawal symptoms, and partially cure addictive behaviors (68–70).

The mesolimbic reward system plays a crucial role in the onset of addiction, exerting influence over dopamine neurons (71). The nucleus accumbens (NAcc), as the core of the reward system, is interconnected with the ventral tegmental area (VTA) (72). Alterations in the nucleus accumbens (NAcc) neural circuitry are pivotal for reward learning, receiving excitatory projections from brain regions such as the prefrontal cortex (PFC) and thalamus (73). Through acupuncture, modulation of endogenous opioid input and dopamine neuron activity within the nucleus accumbens (NAcc) and ventral tegmental area (VTA) upstream neural circuits can be achieved, thereby addressing the motor and emotional abnormalities induced by substance addiction (74). Research suggests that acupuncture at the Shenmen (HT7) can alleviate withdrawal tremors, anxiety, and compulsive drug-seeking behavior in ethanol-dependent rats by activating the arcuate nucleus of the hypothalamus (ARC) to input enkephalins into the nucleus accumbens (NAcc) (75). In studies on cocaine addiction, this team found that acupuncture at Shenmen (HT7) suppressed cocaine-induced motor symptoms via signals mediated by the ulnar nerve A fibers (76). Further investigations suggest that this acupuncture signal may activate the lateral habenula (LHb)-rostromedial tegmental nucleus (RMTg) circuitry via peripheral tactile pathways, ultimately inhibiting dopamine (DA) neurons (77).

During drug withdrawal, compulsive drug-seeking behavior is often accompanied by severe negative emotions such as anxiety and depression (78). The lateral habenula (LHb) plays a crucial role in negatively regulating reward behavior, aversive emotions, and behavioral inhibition (79). Primarily receiving input from the basal ganglia and ventral forebrain, LHb neurons possess the function of inhibiting dopamine neurons (80). Studies have elucidated multiple input pathways to the lateral habenula (LHb), including projections from glutamatergic neurons in the medial prefrontal cortex (mPFC), which elicit aversive responses (81). Addictive substances such as cocaine, nicotine, and ethanol can enhance lateral habenula (LHb) activity, leading to aberrant excitatory and inhibitory synaptic transmission and thus mediating the generation of negative emotions. Augmenting glutamatergic transmission from the medial prefrontal cortex (mPFC) to the lateral habenula (LHb) may be one of the mechanisms by which acupuncture suppresses psychomotor symptoms in cocaine-addicted rats (82). Furthermore, acupuncture can alleviate drug addiction by modulating gamma-aminobutyric acid (GABA) transmission and dopamine expression levels in the ventral tegmental area (VTA) (83). Studies using optogenetics and other techniques suggest that acupuncture may mediate the regulatory effect of GABAergic neurons in the ventral tegmental area (VTA) via activation of central amygdala (CeA), resulting in reduced DA release in the nucleus accumbens (NAcc) and thereby suppressing abnormal motor behavior and positive emotional states induced by methamphetamine (84).

In conclusion, recent research indicates that acupuncture improves symptoms of substance addiction primarily by modulating dopaminergic neural circuits between the prefrontal cortex, thalamus, and the reward systems nucleus accumbens (NAcc) and ventral tegmental area (VTA). Additionally, acupuncture may exert its effects by inhibiting LHb-mediated negative regulation of reward behavior, modulating relevant cortical or reward system inputs to the LHb, suppressing dopamine release in the nucleus accumbens (NAcc) and ventral tegmental area (VTA), and ameliorating substance addiction. Studies have also highlighted the dorsal column pathway as a potential route for acupuncture treatment of cocaine addiction, providing insight into the complete pathway of acupuncture signals from the periphery to the central nervous system.

## 5 Cognitive impairment

Cognitive impairment refers to a state when an individual’s capacity to think, recall, and process information is affected due to various conditions that impact brain function. Mild cognitive impairment (MCI), dementia, Alzheimer’s disease (AD), vascular dementia (VaD), frontotemporal dementia (FD), and traumatic brain injury are frequent causes of cognitive impairment, with Alzheimer’s disease being particularly prevalent. This condition is a degenerative neurological disease characterized by the gradual demise of nerve cells and the deterioration of connections between them in the brain. The initial symptoms typically involve memory loss and cognitive decline. As the disease advances, the patient’s ability to carry out daily activities gradually deteriorates, finally resulting in severe cognitive impairment (85). While a treatment for the condition is not yet available, timely detection and management can effectively decelerate the advancement of the illness and enhance the overall well-being of patients (86). Several recent studies have documented the benefits of acupuncture in the management of Alzheimer’s disease. The mechanism of acupuncture in treating Alzheimer’s disease involves several factors, such as regulating Aβ metabolism and tau protein phosphorylation in a beneficial way, modulating neurotransmitters, reducing damage to synaptic and neuronal function, suppressing neuroinflammatory responses, and enhancing brain energy metabolism (87–89). Thus, acupuncture intervention in Alzheimer’s disease has a multi-target, multi-pathway network modulation impact, which aligns with the overall modulation properties of acupuncture.

The hippocampus stores each experience as a unique memory representation through a mechanism called pattern separation (PS), then retrieves this representation from partial cues via pattern completion (PC) to support memory (90). Impaired pattern separation is often observed in neurocognitive and neurodegenerative disorders. Adult hippocampal neurogenesis and synaptic plasticity in CA1 and CA3 regions are closely linked to pattern separation (91). Furthermore, the medial septum (MS) is involved in central cholinergic (ACh) neuron projections to the hippocampus and entorhinal cortex via the fimbria-fornix, exciting hippocampal pyramidal cells to facilitate learning and memory tasks (91). The M1 receptor plays a crucial role in long-term potentiation (LTP) in the hippocampal CA1 region. Researchers have found that the medial septum (MS)-vertical limb of the diagonal band (MS/VDB)-dentate gyrus (DG) cholinergic neural circuitry may participate in promoting hippocampal neurogenesis and reducing impaired pattern separation by enhancing cholinergic neuron survival, promoting acetylcholine transport, among other mechanisms. Dysfunction in the medial septum (MS)-hippocampal cholinergic system may lead to decreased learning and memory abilities. While, electroacupuncture can improve memory impairment in dementia model mice by activating this circuitry. Researchers have suggested that electroacupuncture may treat early learning and memory impairment caused by diabetes by modulating the medial septum (MS)-hippocampal cholinergic circuitry, inhibiting excessive acetylcholine output from the medial septum (MS) to the hippocampus, enhancing M1 receptor activity, and rectifying nerve growth factor (NGF) and pro-nerve growth factor (proNGF) level imbalances in the medial septum (MS) and hippocampus (92).

Diabetes may also induce this dysfunction, concomitant with impaired hippocampal plasticity and dysregulated levels of pro-nerve growth factor (proNGF) (93). In addition to the hippocampus, research has shown that the dorsal raphe nucleus serotonergic neurons and other upstream brain regions also project to the hippocampus, which is related to cognitive function. This suggests that the hippocampus and upstream brain regions may be key areas for research on cognitive disorders. The neurons in the hippocampus are mainly glutamatergic neurons, which are closely related to the activity of glutamate AMPA receptors, NMDA receptors, and CaMKII, all of which are crucial for the formation of long-term potentiation (LTP). Acupuncture therapy has been shown to improve cognitive function by affecting the hippocampal glutamatergic neuron circuit (94). Although the role of GABAergic neurons in the acupuncture treatment of cognitive disorders has not been fully studied, there is evidence to suggest that inhibition of the activity and function of GABAergic interneurons may lead to structural abnormalities in excitatory synaptic transmission pathways, reducing synaptic plasticity and resulting in cognitive disorders (95). Therefore, GABAergic interneuron-related neural circuits may serve as a new target for research on cognitive disorders.

As a whole, researchers conducted comprehensive assessments of changes in learning abilities in animal experiments before and after electroacupuncture treatment. They used various behavioral tests, including novel object recognition, novel location recognition, Morris water maze, and Y-maze experiments, thereby enhancing the accuracy of cognitive learning ability evaluations. These tests improved the accuracy of evaluating cognitive learning abilities. However, current studies mainly focus on animal spatial memory and short-term memory capabilities, further research is needed to elucidate long-term memory and the hippocampal cholinergic or glutamatergic pathways.

## 6 Gastrointestinal disease

Gastrointestinal diseases are prevalent globally, and acupuncture has a lengthy historical background and extensive clinical expertise in managing such conditions (96, 97). Previous literature has shown that acupuncture is effective in the treatment of functional dyspepsia, functional diarrhea, irritable bowel syndrome with diarrhea, functional constipation, and ulcerative colitis (98–101). The mechanism may be related to acupuncture can regulate the function of gastrointestinal tract, relieve pain, regulate immune function, improve mood, and maintain intestinal microecological balance (96, 102).

Studies have indicated that higher central nuclei associated with the treatment of gastrointestinal diseases include the dorsal vagal complex (DVC), paraventricular nucleus (PVN), and central amygdala (CeA). Within the brainstem, the dorsal vagal complex (DVC) is considered a critical region for regulating gastrointestinal smooth muscle movement and glandular secretion (103). It connects bidirectionally the nucleus tractus solitarius (NTS) and the dorsal motor nucleus of the vagus nerve (DMV) (104). It is speculated that gastrointestinal sensory instructions may reach the dorsal motor nucleus of the vagus nerve (DMV) or nucleus tractus solitarius (NTS) directly or indirectly via the vagus nerve. After signal integration in these regions, the dorsal motor nucleus of the vagus nerve (DMV) communicates the information through the vagus nerve to higher central nuclei, thereby regulating gastrointestinal activity and glandular secretion (105). Studies have found that electroacupuncture at the Zusanli (ST36) point may regulate gastrointestinal hormone levels and affect gastrointestinal motility via the DVC-vagus nerve-stomach pathway (106). Another study has indicated that acupuncture is involved in the PVN-DVC-vagus nerve pathway, influencing gastric motility (107). The hypothalamus influences gastrointestinal movement through the autonomic nervous system, parasympathetic nervous system, and endocrine regulation. The cerebellum, besides participating in motor coordination, also plays a role in regulating visceral activity, thus affecting gastrointestinal motility in synergy. Through fMRI, it has been observed that acupuncture at the Zusanli (ST36) and Tianshu (ST25) points enhances neural signals in the right cerebellar cortex and bilateral hypothalamus, suggesting involvement of the cerebellum-hypothalamus circuit in regulating gastrointestinal function along the stomach meridian (108). Another study found that acupuncture at Zusanli (ST36) may improve gastrointestinal function via the lateral hypothalamic area (LHA)-dentate nucleus (DN) cerebellar circuit (109). These studies suggest that acupuncture may exert its effects on gastrointestinal motility through different neurons within the hypothalamus-cerebellum circuit. Further research indicates that the paraventricular nucleus (PVN) receives projections from GABAergic neurons in the central amygdala (CeA), and electroacupuncture at the Zusanli (ST36) point may regulate appetite, promote gastric emptying, and thus improve gastrointestinal function through the CeA^GABA^-PVN circuit (110).

From the above studies, the neurological pathways of acupuncture treatment for gastrointestinal diseases mostly concentrates on the brainstem, hypothalamus, and cerebellum. These regions are responsible for regulating visceral activity. Acupuncture can enhance gastrointestinal function by influencing the action of digestive smooth muscles through the regulation of several gastrointestinal hormones within neural circuits. Researchers can employ advanced methods like fMRI, neural tracing, and chemogenetics to detect the activation of specific brain areas and nuclei, such as the hypothalamus, cerebellum, and central amygdala (CeA), along with their projections, during acupuncture treatment for gastrointestinal illnesses. However, in order to conduct a more accurate examination of the exact activities of neurons within the neural circuitry that is affected by acupuncture, and to gain a more complete understanding of its central mechanisms, additional study using advanced techniques like optogenetics may be required.

## 7 Other diseases

Furthermore, acupuncture has been shown to affect the circuitry mechanisms of post-stroke functional impairments, chronic itch, comorbid anxiety and depression, and sleep disorders. Recent studies suggest that electroacupuncture at Baihui (GV20) and Dazhui (GV14) points may improve blood circulation in the contralateral primary somatosensory cortex (S1)—motor cortex (M1) circuit, enhancing the activity of neurons in this circuit and thereby promoting the recovery of limb motor function after unilateral cerebral infarction (111). Additionally, the paraventricular nucleus of the hypothalamus (PVN) may be involved in electroacupuncture treatment for post-stroke dysphagia (PSD) and is associated with fiber connections between the parabrachial nucleus (PBN) and nucleus tractus solitarius (NTS) (112). Further research indicates that electroacupuncture at Lianquan (CV23) point may treat PSD through the M1-PBN-NTS neural circuit by reducing the activation of contralateral M1/L5 on parabrachial nucleus (PBN) and nucleus tractus solitarius (NTS) (113). Moreover, electroacupuncture treatment for chronic itch may be associated with the expression of CB1 receptors in the descending inhibitory system. High-frequency and high-intensity electroacupuncture reduce the expression of CB1 receptors in GABAergic neurons of the ventrolateral periaqueductal gray (vlPAG), inhibit serotonin (5-HT) levels in the spinal cord, and thereby alleviate chronic itch (114). Additionally, the mesolimbic reward system circuitry not only participates in acupuncture analgesia and addiction treatment but has also been found to play a role in acupuncture treatment for comorbid anxiety and depression and sleep disorders. For example, acupuncture can treat comorbid anxiety and depression by modulating neural adaptation in the brain reward circuitry of atopic dermatitis mice (115). Moreover, Xi et al. (116) found that electroacupuncture intervention at the Yintang (GV29) and Shenting (GV24) points resulted in decreased dopamine (DA) levels in the ventral tegmental area (VTA) and nucleus accumbens (NAcc), along with reduced expression of dopamine receptor 1 (D1R) and increased expression of dopamine receptor 2 (D2R) in the nucleus accumbens (NAcc) in a chronic sleep deprivation model in rats. These findings suggest that electroacupuncture at the Yintang and Shenting points may treat chronic insomnia through the VTA-NAc dopamine (DA) circuit. Additionally, Zhu et al. (117) proved that electroacupuncture at Zusanli (ST 36), Fenglong (ST 40), Guanyuan (CV 4), and Zhongwan (CV 2) effectively ameliorated insulin resistance via activating NTS^GLP-1^-VTA^DA^ circuit.

## 8 Summary and future outlook

**Figure 1:**
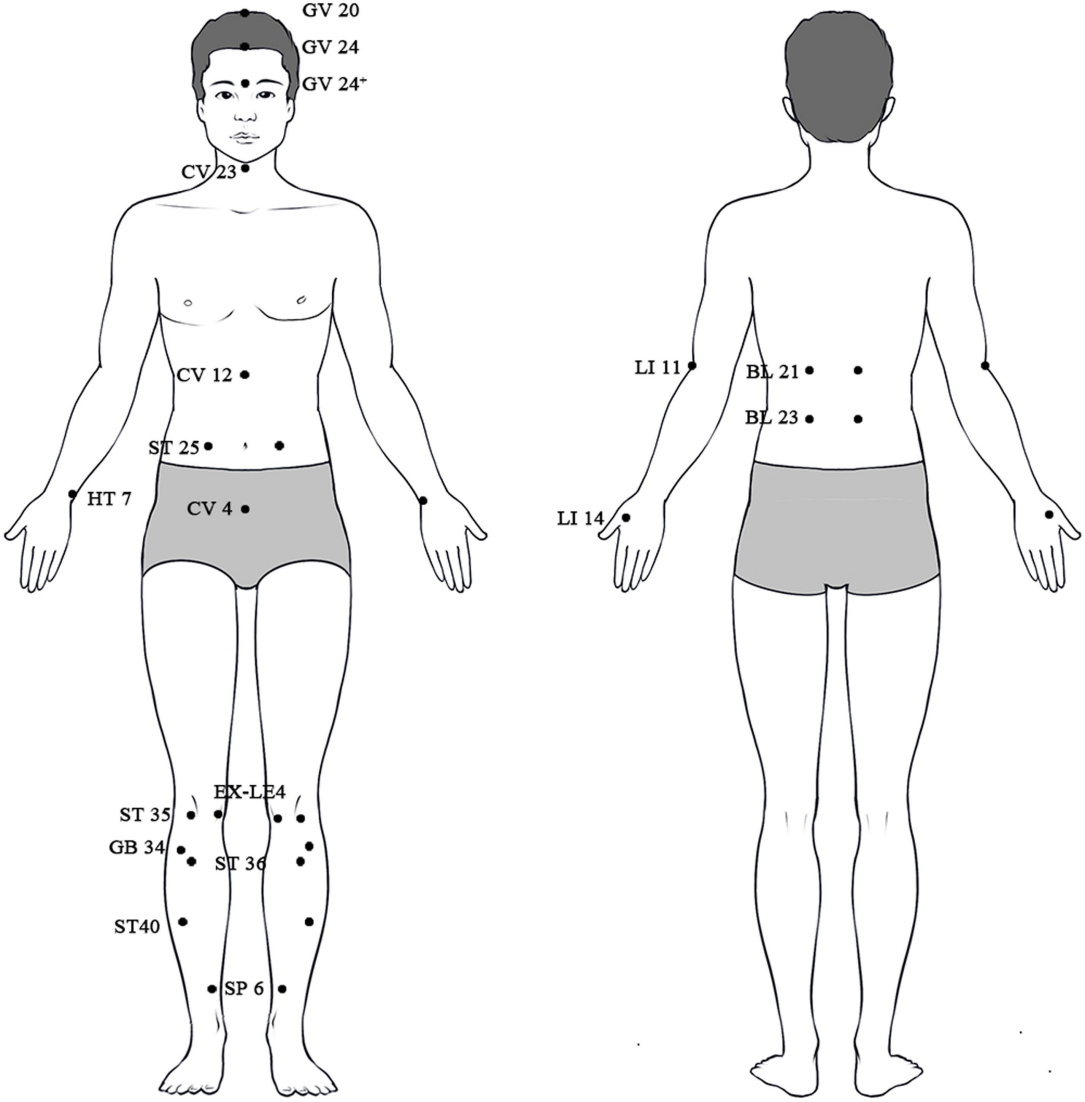
The locations of all the mentioned acupoints on the human body.

Recent research on acupuncture pathways has mostly focused on pain and its related problems, including anxiety, Parkinson’s disease (PD), addiction disorders, cognitive dysfunction, gastrointestinal diseases, and other afflictions. Out of them, research on pain modulation pathways has been more common. We have conducted research that covers a wide range of topics, including the sensitization of nociceptive pathways, the mechanisms of endogenous pain modulation systems, and the modulation pathways involving the thalamus, cortex, and mesolimbic reward systems at various levels. Various brain regions and nuclei have different impacts on the perception of pain. For example, the periaqueductal grey (PAG) and rostral ventromedial medulla (RVM) can modify the transmission of pain signals in the spinal cord to suppress pain. On the other hand, the thalamus, anterior cingulate cortex (ACC), and prefrontal cortex (PFC) regulate the emotional and attentional aspects of pain. These structures interact through projections of many types of neurons, including GABAergic, glutamatergic, and serotonergic neurons, to either enhance or suppress the feeling of pain and emotion. Acupuncture can influence these pathways, indicating that acupuncture signals can alleviate pain by targeting several objectives at different phases of pain perception, affecting diverse locations and nuclei in the brain. The relative positions of all the mentioned acupoints on the human body are shown in Figure 1.

Research indicates that acupuncture is largely effective in treating motor symptoms of Parkinson’s disease (PD) by improving balance in the basal ganglia and thalamocortical circuits. Furthermore, this suggests the importance of identifying alternative pathways, such as the corticothalamic circuit (CTC), that can compensate for the decreased basal ganglia pathways in order to improve the effectiveness of acupuncture in treating Parkinson’s disease. Acupuncture has the ability to regulate the activity of dopaminergic neural circuits in specific areas of the brain, such as the prefrontal cortex, thalamus, and reward systems. This can be used as a treatment for substance addiction. Acupuncture in cognitive impairment therapy primarily affects the cholinergic neuronal pathways connecting the medial septum (MS) and diagonal band (DB) to the hippocampus. It also influences the glutamatergic neuronal circuitry within the hippocampus. The neurological pathways primarily engaged in acupuncture treatment for gastrointestinal illnesses include the brainstem, hypothalamus, and cerebellar circuitries, as well as the dorsal vagal complex route.

Overall, the aforementioned studies have utilized modern techniques such as neural tracing, chemogenetics/optogenetics, and functional brain imaging (fMRI), combined with behavioral testing, to demonstrate specific neural projections, regulate or precisely regulate specific cells within circuits, and depict neural signal responses before and after acupuncture in brain areas or nuclei, enhancing the comprehensiveness and objectivity of acupuncture pathway research. They have mainly explored the regulatory effects of acupuncture on specific neuronal projections within the central nervous system in the aforementioned diseases, with some studies also involving research on the peripheral sensory level transmission pathways. This aids in elucidating the central nervous system effects of acupuncture therapy, suggesting that acupuncture may exert its therapeutic effects by modulating neural circuit mechanisms that exhibit functional differences in various physiological and pathological states.

Neural pathways are crucial focal points for studying acupuncture effects; however, there are still some issues to address. Firstly, due to limitations in tracing technology and techniques like chemical genetics/optogenetics, research subjects are predominantly animal models, with varied standards for constructing these models. Clinical studies are relatively limited, with most having small sample sizes. It is hoped that future advancements in technology will facilitate large-sample, multicenter clinical studies to enhance the reliability of conclusions. Secondly, in experimental animals, electroacupuncture is usually the preferred method, and male mice are predominantly used as subjects. Does gender affect circuitry mechanism research? In clinical research, are there differences between needling on the left and right sides due to handedness? It is hoped that future research will strengthen the standardization of acupuncture, promote standardized acupoint selection and operation procedures, and further investigate these differences to enhance the objectivity of experimental results. Thirdly, central nervous system circuitry research currently predominates, with a limited number of diseases being studied. Therefore, it is hoped that future research will gradually expand the scope of disease research on acupuncture pathways, improve the comprehensive study of acupuncture effects on complete pathways from the periphery to the central nervous system, and then to target organs.

As the aforementioned concerns are gradually resolved, future research on the neural circuit mechanics of acupuncture is expected to become more thorough and comprehensive. Specifically, it is necessary to broaden the range of disease research and thoroughly investigate the acupuncture pathway mechanisms of beneficial diseases. This will enhance our comprehension of the neurobiological impacts of acupuncture therapy and direct clinical practice towards more efficient treatments.

## Author contributions

XW: Writing – review & editing, Writing – original draft, Resources, Investigation, Conceptualization. JW: Writing – review & editing, Writing – original draft, Resources, Investigation, Conceptualization. RH: Writing – original draft, Resources, Investigation. CY: Writing – review & editing, Writing – original draft, Supervision, Resources, Funding acquisition, Conceptualization. FS: Writing – review & editing, Supervision, Resources, Funding acquisition, Conceptualization.
