# Corrigendum: Neural circuit mechanisms of acupuncture effect: where are we now?

**DOI:** 10.3389/fneur.2025.1576213

**Published:** 2025-02-28

**Authors:** Xuesong Wang, Jia Wang, Rui Han, Chaochao Yu, Feng Shen

**Affiliations:** ^1^College of Acupuncture-Moxibustion and Tuina, Hebei University of Chinese Medicine, Shijiazhuang, China; ^2^Department of Acupuncture and Moxibustion, Wuhan Hospital of Integrated Traditional Chinese and Western Medicine, Wuhan, China; ^3^Department of Child Rehabilitation Medicine, Qujing Hospital of Maternity and Childcare, Qujing, China; ^4^Department of Tuina, Shenzhen Traditional Chinese Medicine Hospital, Shenzhen, China; ^5^The Fourth Clinical College of Guangzhou University of Chinese Medicine, Shenzhen, China; ^6^Department of Rehabilitation, Union Hospital, Tongji Medical College, Huazhong University of Science and Technology, Wuhan, China; ^7^College of Acupuncture and Orthopedics, Hubei University of Chinese Medicine, Wuhan, Hubei, China

**Keywords:** acupuncture, neural circuit mechanisms, pain, Parkinson's disease, addictive disorders, cognitive disorders, gastrointestinal disorders, review

In the published article, there was an error in the **Funding statement**.

The statement was previously written as, “This work was funded by Shenzhen Science and Technology Program (No. 2021- 22154); National Natural Science Foundation of China (No. 82205271); Key Chinese Medicine Project supported by Hubei Provincial Natural Science Foundation (2023AFD113); Chinese Medicine Research Project supported by Hubei Administration of Traditional Chinese Medicine, China (No. ZY2023F138).,” with the project number stated for ‘Shenzhen Science and Technology Program’ given incorrectly as ‘(No. 2021- 22154)’. The correct Funding statement appears below.

